# Liposomes against Alzheimer’s Disease: Current Research and Future Prospects

**DOI:** 10.3390/biomedicines12071519

**Published:** 2024-07-08

**Authors:** Christiana Constantinou, Katerina Meliou, Athanasios Skouras, Panoraia Siafaka, Panayiota Christodoulou

**Affiliations:** 1 Department of Life Sciences, School of Sciences, Pharmacy Program, European University Cyprus, 2404 Nicosia, Cyprus; cc181708@students.euc.ac.cy (C.C.); km181489@students.euc.ac.cy (K.M.); p.siafaka@euc.ac.cy (P.S.); 2 Department of Nursing, Faculty of Health Sciences, Hellenic Mediterranean University, 71004 Heraklion, Crete, Greece; nasosskouras@upatras.gr; 3 School of Medicine, European University Cyprus, 2404 Nicosia, Cyprus

**Keywords:** Alzheimer’s disease, therapy, liposomes, functionalization, targeting, brain, nanoparticles

## Abstract

Alzheimer’s disease, the most common neurodegenerative disease, affects more than 60 million people worldwide, a number that is estimated to double by 2050. Alzheimer’s disease is characterized by progressive memory loss, the impairment of behavior, and mood changes, as well as the disturbed daily routine of the patient. Although there are some active molecules that can be beneficial by halting the progression of the disease, the blood–brain barrier and other physiological barriers hinder their delivery and, consequently, the appropriate management of the disease. Therefore, drug delivery systems that effectively target and overcome the blood–brain barrier to reach the targeted brain area would improve treatment effectiveness. Liposomes are lipophilic carriers that consist of a phospholipid bilayer structure, simulating the physiological lipidic layer of the blood–brain barrier and enabling better delivery of the drug to the brain. Given that pure liposomes may have less targeting affinity than functionalized liposomes, modification with groups such as lactoferrin, poly(ethylene glycol), and transferrin may improve specificity. In this mini-review, we summarize the literature on the use of liposomes for the treatment of Alzheimer’s disease, focusing on the functionalization moieties of liposomes. In addition, challenges in brain delivery are also discussed.

## 1. Introduction

Alzheimer’s disease (AD), the most devastating neurodegenerative disease, affects the central nervous system (CNS), leading to memory alterations, progressive cognitive neuronal dysfunction, and behavior impairment, as well as poor quality of life [1]. Despite the recent advancements in the diagnosis and treatment of AD, more effective treatments are required. Today, an estimated 6.7 million Americans 65 years of age and older suffer from AD. If medical advancements are not made to prevent, halt, or cure AD, this figure may rise to 13.8 million by 2060. Moreover, the prevalence of dementia is expected to triple globally and double in Europe by 2050 [2]. The main obstacles hindering drug efficacy are physiological barriers, such as the blood–brain barrier (BBB) and blood–cerebrospinal fluid (CSF) barrier, as well as unknown pathophysiologic mechanisms that lead to neurodegeneration [3,4].

The brain regions in charge of memory, language, and thought processes are the first to suffer neuronal degeneration in AD. Therefore, thinking, language, and memory issues are frequently the initial symptoms. Abnormal changes in brain function are believed to start at least 20 years prior to the onset of symptoms, which are progressive and irreversible [5]. The affected abilities and the rate of progression differ from person to person. AD eventually causes neuronal loss in areas of the brain responsible for fundamental body activities, including walking and swallowing, leading to the patient becoming bedridden and needing 24 h care. According to studies, patients who are diagnosed with Alzheimer’s dementia at 65 years of age or beyond typically live for 4 to 8 years; however, some may live up to 20 years [6].

AD demonstrates two main prototypical lesions: senile plaques (SPs) formed by the assembly of β-amyloid protein (Aβ) and neurofibrillary tangles (NFTs) consisting of phosphorylated tau protein (P-tau) aggregates. β-Amyloid protein can be deposited in capillary walls, arteries, and arterioles, resulting in amyloid cerebral angiopathy. This phenomenon can lead to vascular wall component degeneration and can obstruct blood flow [7]. P-tau belongs to the family of microtubule-associated proteins (MAPs) and has an impact on neuronal polarization, axonal transport and growth, and normal neuronal function. Structurally, P-tau consists of four distinct segments: the N-terminal segment, the proline-rich domain (PRR), the microtubule-binding domain (MTBD), and the C-terminus. The MTBD of P-tau is very important, as it facilitates its interaction with, and thus stabilizes, microtubules. The stabilization of microtubules by P-tau is critical for the regulation of neuronal activity, synaptic plasticity, neurogenesis, and other necessary processes for normal neuronal function. Moreover, these proteins encourage dendritic structure and axonal transit [8,9]. Furthermore, P-tau can self-polymerize, resulting in neuronal damage [10,11]. P-tau can interact with other proteins and become phosphorylated. In AD, tau becomes hyperphosphorylated, leading to reduced affinity with microtubules, thus promoting their aggregation and the formation of neurofibrillary tangles. This results in a decrease in the functionality of the neurons and eventually their apoptosis [12,13]. Pathological alterations in P-tau, resulting in the development of NFTs and paired helical filaments, are hallmarks of these neurological disorders and can be used as a therapeutic target [9].

Despite the identification of various possible mechanisms leading to AD, available pharmacological management includes basic drug molecules that have been used for many years. More specifically, the mainstay drugs for AD are inhibitors of acetylcholinesterase (AChE), the enzyme that breaks down acetylcholine (Ach). AChE inhibitors such as Tacrine, Donepezil, Rivastigmine, Galantamine, and the N-methyl-D-aspartate (NMDA) receptor antagonist Memantine have been approved by the Food and Drug Administration for AD management [3,4,14,15]. The aforementioned drugs cannot halt AD progression but can only manage the symptoms. In fact, they have low therapeutic efficacy, and some of them are non-selective and cause hepatotoxicity [16]. The main obstacle hindering the development of new treatments for neurodegenerative disorders, including AD, is the delivery of drugs across the BBB and the cerebrospinal fluid barrier, which divides blood from CSF. Nanotechnology-based approaches may be able to overcome this issue and improve neurodegenerative disorder therapy.

The BBB is an integrated cellular barrier composed of brain endothelial cells and perivascular components, which prevents chemicals from entering the brain from the blood. Drugs can be delivered to different brain cells, such as neurons, astrocytes, oligodendrocytes, and microglia, using vehicles, such as liposomes [17]. This is a potential strategy for a more targeted delivery of drugs to cells involved in the pathophysiology of AD. Liposomes have important benefits for drug delivery to the brain, including biocompatibility, high stability, and the ease of attachment of ligands for receptor-mediated drug delivery. This review explores liposomes, with a focus on their brain delivery mechanisms and therapeutic approaches targeting AD pathology. The authors have performed a thorough literature search using keywords such as Alzheimer’s disease, therapy, liposomes, functionalization, targeting, brain, nanoparticles (NPs), and nanocarriers.

**Figure 1:**
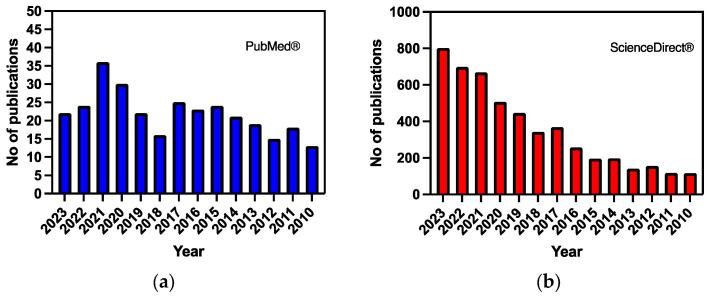
The number of articles derived from the search for Alzheimer’s disease AND liposomes based on data from (**a**) PubMed^®^ and (**b**) ScienceDirect^®^.

The databases used were PubMed^®^ and ScienceDirect^®^. According to our inclusion criteria, all studies that included the previous terms, especially Alzheimer’s disease AND functionalized liposomes, were added. Figure 1 depicts the number of articles derived by searching the aforementioned databases.

## 2. Challenges in Brain Delivery

Neurological and neurodegenerative disease treatments can be significantly enhanced by the efficient delivery of therapeutic medicines to the brain in order to affect specific cells or regions of the brain. Even though the BBB is extremely specific about what can go through, gaps can nonetheless exist. Certain tiny lipophilic substances can move between the circulation and the CNS by passively diffusing through endothelial cells [18]. The BBB is permeable to water and very small hydrophilic and charged molecules through the process of paracellular transport [19]. Nevertheless, membrane barriers prevent larger molecules from passing through [20]. The BBB, therefore, makes pharmacotherapy for CNS illnesses more difficult since it prevents all macromolecular therapies and more than 98% of small-molecule medications from entering the brain, thus limiting their effectiveness [21,22]. Drugs that are not delivered sufficiently to the brain accumulate in other organs and tissues, which exacerbates side effects and reduces therapeutic efficacy.

Efflux transporters present at the BBB also actively pump many therapeutic agents back into the bloodstream, reducing their concentration in the brain. The ATP-Binding Cassette Subfamily B Member 1 (ABCB1) gene, sometimes referred to as multidrug resistance 1 (MDR1), produces P-glycoprotein (P-gp), which pumps different drugs out of cells and back into the bloodstream through ATP-dependent efflux pumps [23,24]. Therefore, the inhibition of P-gp could lead to the inhibition of efflux at the BBB, resulting in increased brain distribution and bioavailability of the substrate drug [25,26]. CNS drugs are also metabolized by BBB-expressed enzymes, leading to their inactivation. Patient-specific polymorphisms and variations in the expression of these enzymes may lead to differences in the efficacy of CNS medications [27].

Systemically administered drugs can be distributed widely throughout the body, leading to the low efficiency of their delivery to the brain and potential systemic toxicity [28]. Since nanotechnology can decrease the negative side effects associated with nonspecific drug distribution, increase drug concentration at the desired site of action, and ultimately improve therapeutic effectiveness, it is a crucial tool for developing new systems for the efficient delivery of potential therapeutic and diagnostic compounds to specific areas of the brain [29].

## 3. Overview of Liposome Technology

**Figure 2:**
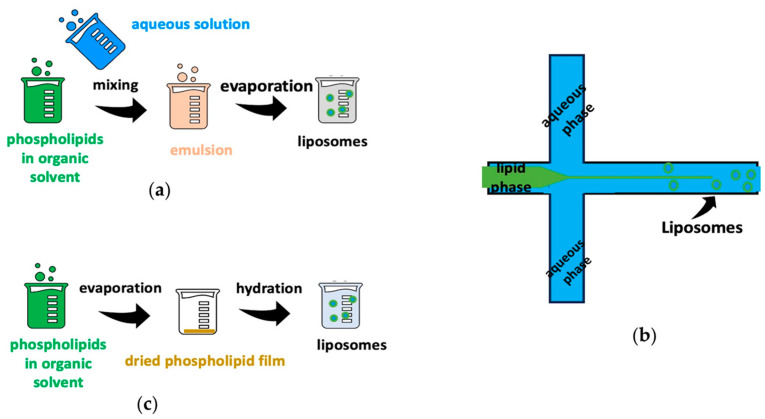
Several liposome preparation methods: (**a**) reverse-phase evaporation method, (**b**) microfluidic method, and (**c**) film hydration method.

Liposomes are spherical vesicles and are characterized as amphiphiles, as they consist of a hydrophilic core surrounded by a phospholipid bilayer. They can be synthesized in different sizes, with different types of phospholipids, and with different preparation procedures, such as the reverse-phase evaporation method, microfluidic method, and film hydration method (Figure 2). Also, the outer surface of liposomes can contain one or more phospholipids layers, while various molecules can be added that enhance their properties [30,31,32].

**Figure 3:**
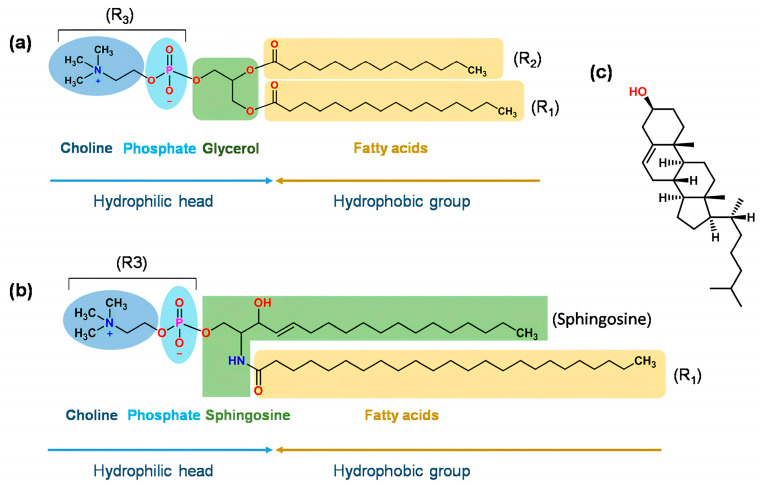
(**a**) Structure of glycerolphospholipid. R1 and R2 can be saturated or unsaturated fatty acids, such as decanoic acid, lauric acid, palmitic acid, oleic acid, myristic acid, stearic acid, or erucic acid. R3 can be phosphatidylcholine (PC), phosphatidyl ethanolamine (PE), phosphatidyl serine (PS), phosphatidyl inositol (PI), phosphatidic acid (PA), phosphatidylglycerol (PG), or cardiolipin. (**b**) Structure of sphingomyelin. (**c**) Structure of cholesterol. Obtained from Ref. [34].

Phospholipids structurally consist of a polar and a non-polar segment. On the one hand, the non-polar part is represented by the hydrophobic tails of fatty acids, and on the other hand, in the polar part is the hydrophilic head containing choline, a phosphate group, and glycerol (Figure 3). Thus, amphiphilic molecules are formed that react according to the environment that exists around them. When phospholipids are in an aqueous environment, they are rearranged in order to minimize the interactions between the hydrophobic part and the aqueous medium. This spontaneous formation of liposomes has sparked interest in its use as a nanocarrier [33].

For these reasons, liposomes are able to provide stable encapsulation of various drugs, either lipophilic or hydrophilic, due to their amphiphilic nature and transport them into the body with great efficiency. In addition, due to the fact that they are composed of biomolecules found in the body, they are non-toxic, have good compatibility, and do not activate the immune system. However, the functionalization of liposomes with molecules, such as polyethylene glycol (PEG), may increase their immunogenicity [35,36].

## 4. Strategies for Increasing the BBB Penetration of Liposomes

**Figure 4:**
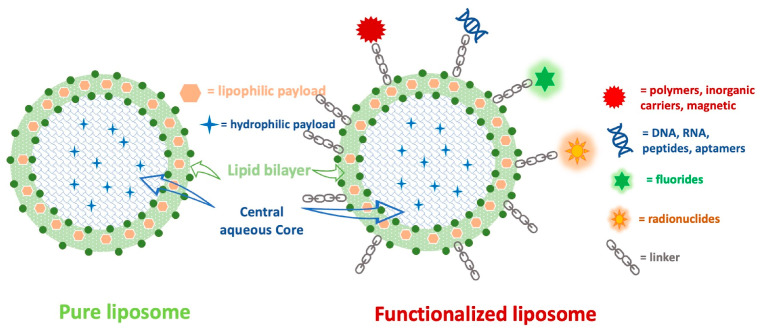
The structural differences between pure and functionalized liposomes.

The functionalization of liposomes by modifying their surfaces with polymers, inorganic carriers, fluorides, DNA, RNA, peptides, magnetic nanoparticles, and radionuclides can improve their specificity and therapeutic outcomes. Figure 4 depicts the differences between a pure liposome and a functionalized liposome.

The structure and composition of liposomes, which resemble those of the cell membrane, lead to their reduced toxicity [33]. Furthermore, their lipophilicity facilitates their passive diffusion across the BBB. Liposomes are prepared from natural as well as synthetic lipids, including predominantly phosphatidylcholine and cholesterol. Phosphatidylcholine becomes hydrogenated, increasing the liposome’s physicochemical stability, specifically at transition-phase temperatures, at which phospholipids change from a gel to a liquid crystalline phase [37]. This property is an important advantage during the preparation step of the nanocarrier [38,39]. Cholesterol molecules interact with both the polar and non-polar parts of phospholipids, forming phospholipid bilayers that provide stability and rigidity to the liposome. Cholesterol also reduces the permeability of water-soluble molecules through the membrane. In addition, cholesterol can retain the bioactive molecule in the liposome core while at the same time preventing its release into the blood plasma [40], as it provides mechanical stiffness to the membrane [39].

### 4.1. Adsorptive-Mediated Transcytosis (AMT)

AMT is one of several pathways that enable substances to cross the BBB. This particular method involves the adsorption of molecules to the endothelial cell surface of the BBB through nonspecific interactions, such as electrostatic forces. Once the molecules are adsorbed on the cell surface, they can be internalized by the endothelial cells and transported across the cell body to be released on the other side, thus crossing the BBB [41].

To this end, Simonis et al. compared the transport properties of cationic liposomes, incorporating gemini amphiphiles with varying stereochemistry, with those of neutral liposomes in an in vitro human BBB model. This approach aimed to elucidate how the structural nuances of liposomes influence their ability to cross the BBB. The results indicated that the stereochemistry of gemini amphiphiles significantly affects the passage of cationic liposomes, showcasing a marked difference in the transport efficiency between cationic and neutral liposomes. However, the employment of cationic nanocarriers for brain delivery presents a significant limitation: the nonspecific absorption by tissues outside the brain and the interaction with serum proteins, which diminish their surface charge, necessitate the use of substantial quantities of these nanocarriers to achieve therapeutic effectiveness, potentially increasing the risk of cytotoxicity [42].

In a different approach, Joshi et al. examined how the surface charge of liposomes affects their uptake in the brain by administering differently charged liposomes into rats’ carotid arteries under reduced blood flow conditions, with and without altering the BBB permeability using ultrasound. By utilizing an intra-arterial injection, plasma protein interactions as well as nonspecific uptake were minimized. The findings suggest that cationic liposomes significantly enhance brain deposition, showing much higher peak and end concentrations compared to neutral and anionic liposomes. This indicates that for intra-arterial delivery, especially in treating brain tumors, the surface charge of liposomes is crucial, even without BBB disruption [43].

### 4.2. PEGylated Liposomes

When single liposomes are introduced into the bloodstream, plasma proteins coat the liposome membrane, resulting in the formation of a protein corona. These proteins are recognized by the immune system and are phagocytosed, removing them from the circulation. To avoid this problem, PEG, a hydrophilic polymer, is incorporated into the lipid components of the outer liposome membrane. Such modified liposomes containing PEG are called stealth liposomes. The reason that PEG is added is to enhance the surface properties of the liposomes, providing high hydration, thus increasing the hydrodynamic volume and forming a liquid cloud around the polymer. The half-life of PEG-conjugated liposomes depends on the characteristics of PEG, such as the length and concentration of polymer chains on the liposome surface [44]. Moreover, PEG-linked liposomes have increased solubility and resistance to plasma proteins, limiting the formation of protein coronas, preventing phagocytosis, and thus increasing the half-life of the drug [30,45]. An additional advantage of PEG is its lack of toxicity, as well as its good excretion from the body [45].

In general, PEG presents many useful and attractive properties for nanotechnology since it is biocompatible and can be dissolved in either aqueous or organic media. The addition of PEG to the surface of liposomes can be achieved in several ways, such as the physical absorption of the polymer on the surface of vesicles during liposome preparation, the incorporation of PEG–lipid conjugates, or the covalent attachment of reactive groups to the surface of preformed liposomes [46].

### 4.3. Bypassing the BBB: Intranasal Delivery

The nose-to-brain drug delivery method involves the transport of drugs from the nasal mucosa directly to the brain via the olfactory and trigeminal nerve pathways, bypassing the BBB. This route potentially increases treatment efficacy for neurological disorders while reducing the systemic side effects and drug degradation that commonly occur with oral or intravenous administration [47].

In this context, Vasileva et al. prepared mitochondria-targeted liposomes co-loaded with alpha-tocopherol and donezepil hydrochloride as a means of targeting Aβ plaques in the brain through the nose. After the optimization of the liposomal carrier, in vivo experiments were carried out in an AD mouse model. Intranasal liposome administration improved memory impairment, almost to the level of healthy animals, while also slowing Aβ plaque formation, indicating the therapeutic potential of this approach [48].

In another study by Li et al., hydroxy-α-sanshool (HAS), a potential AD treatment, was encapsulated in liposomes in order to enhance its stability. The optimized liposomal formulation demonstrated no nasal mucosal toxicity, in contrast to the HAS solution, and was further used for in vivo experiments in D-Galactose-treated mice. HAS–liposomes demonstrated significant improvements in learning and memory in the AD mouse model, comparable to those of donezepil-treated mice [49]. Saka et al. utilized nose-to-brain liposomal drug delivery in order to enhance imatinib mesylate’s (IMB) brain bioavailability. Optimized liposomal IMB showed promising in vitro safety profiles and significantly improved brain bioavailability in vivo. More specifically, liposomal IMB exhibited a seven times higher area under the curve (AUC) in the brain than the corresponding solution. Furthermore, liposomal IMB showed a higher Tmax, consistent with controlled drug release [50]. Li et al. explored the impact of the intranasal administration of flexible liposomes containing galanthamine hydrobromide (GH) on acetylcholinesterase activity and GH pharmacokinetics in the rat brain. Enhanced acetylcholinesterase inhibition was noted, particularly with intranasal GH in flexible liposomes, compared to oral administration. Pharmacokinetic assessments revealed significant improvements in GH’s Cmax (3 times higher than oral GH) and AUC, with a notable 25% reduction in Tmax with intranasal delivery in comparison with the GH solution [50].

Brain-targeting liposomes modified with the c(RGDyK) peptide to deliver HI-6 directly to the brain, overcoming the BBB, were used to treat organophosphorus compound poisoning. These liposomes showed high encapsulation efficiency and were effective in crossing the BBB in both in vitro and in vivo models. Both intranasal and intravenous delivery routes were utilized, with both having higher acetylcholinesterase reactivation rates compared to free HI-6. Notably, the nasal administration of these liposomes proved more efficacious than intravenous injection in reactivating the enzyme in the brain, offering a promising non-invasive treatment method for organophosphorus poisoning [51].

Narayan et al. explored risperidone encapsulation in liposomes for schizophrenia treatment in rats, comparing three formulations with varying surface charges. Intranasal administration showed lower peak concentrations and almost six times higher Tmax in the systemic circulation compared to intravenous free-drug delivery but enhanced plasma drug exposure, suggesting sustained absorption. Notably, the results were reversed in the brain, with all three preparations presenting higher Cmax and faster Tmax in comparison to a solution administered intranasally. DSPE-PEG liposomes, due to PEGylation and a subsequently longer circulation time, exhibited superior plasma exposure and brain bioavailability. These findings indicate that PEGylated liposomes could effectively transport drugs across the BBB and enhance brain delivery through intranasal administration [52].

### 4.4. Overcoming Efflux Transporters

As stated above, efflux transporters at the BBB actively reduce the concentration of drug molecules in the brain. To this end, strategies to co-deliver efflux transporter inhibitors with the therapeutic agent have been employed.

In this context, RGD-modified liposomes co-encapsulating vinorelbine as a cytostatic agent and tetrandrine as an inhibitor of P-gp have been used as a potential treatment for brain glioma. Optimized liposomal formulations exhibited high encapsulation efficiency, controlled release, and high uptake in both C6 and resistant C6 glial cells. In vivo studies showed an increase in AUC and Tmax for RGD-modified vinorelbine plus tetrandrine liposomes compared to free vinorelbine, while the median survival time was significantly longer than with saline treatment. These results can be attributed to enhanced BBB crossing and P-gp inhibition, as well as the enhanced activation of apoptotic proteins [53].

In another study, Yang et al. prepared verapamil and riluzole cocktail liposomes, utilizing verapamil as a P-gp inhibitor to increase the riluzole brain concentration. The results highlighted that verapamil cocktail liposomes significantly inhibited P-gp levels in a dose- and time-dependent manner, showcasing their potential to facilitate the transport of riluzole to the brain. Liposomal riluzole’s uptake was almost twice that of the free drug, indicating the liposomes’ capability to counteract P-gp-mediated drug resistance [54].

Liposomal formulas containing elacridar and tariquidar have been used to bypass P-glycoprotein at the BBB. Conventional liposomes showed rapid clearance and minimal brain distribution of loperamide. PEGylated liposomes increased the half-lives of elacridar and tariquidar but did not improve their brain penetration or affect loperamide distribution. OX26 F(ab′)2-conjugated PEGylated liposomes, however, enhanced the brain uptake of these drugs and loperamide, without altering loperamide’s plasma pharmacokinetics or liver distribution [55].

## 5. Modifications for Targeted Delivery of Liposomes

The modification of liposomes by conjugation with various ligands that facilitate BBB penetration, such as transferrin and Apolipoprotein E (ApoE), allows the enhanced delivery of drugs to specific locations. An emerging approach for targeted delivery of drugs to the brain is the use of magnetic nanoparticles.

### 5.1. Transferrin–Liposomes

Transferrin (Tf) is an iron-binding blood plasma glycoprotein and the most studied ligand of the transferrin receptor (TfR). TfR is a well-characterized receptor for targeting the BBB and is extensively expressed on the plasma membrane of neurons [56]. Since TfR is more abundant in the capillary endothelium of the BBB than in other organs, it is currently a target for improved drug and gene delivery to the brain.

The brain delivery of the nerve growth factor (NGF) gene using liposomes conjugated to Tf and penetratin, a peptide enhancing cell penetration, was studied in an in vitro co-culture model of the BBB and in a mouse model of AD. These liposomes showed efficient crossing of the BBB and increased the expression of NGF and the presynaptic marker synaptophysin in primary neuronal cells. Moreover, it reduced soluble and insoluble Aβ peptide levels and increased the expression of synaptic markers in the in vivo model, suggesting Tf-conjugated liposomes as a promising vehicle for therapeutic gene delivery to the brain [57].

Tf-conjugated liposomes loaded with vitamin B12 have been used to target the BBB and neuronal cells, which overexpress TfR. In this study, liposomes showed a desirable size of less than 200 nm and a neutral zeta potential, which is appropriate for brain delivery. Vitamin B12 has attracted interest due to its anti-amyloidogenic properties demonstrated in vitro; however, its hydrophilicity and high molecular weight have hindered its application in AD. A recent study demonstrated that the use of Tf-modified liposomes allowed targeted delivery to the brain and the sustained release of vitamin B12 for 9 days. The results of this study showed that the liposomes delayed the formation of Aβ fibrils by inhibiting Aβ fibrillation and disaggregating preformed fibrils. Considering the above, functionalized liposomes are quite promising, and in vivo studies should be performed to assess their therapeutic efficacy [58]. Tf-modified liposomes can also include other molecules that provide additional therapeutic and cytoprotective properties, such as anti-inflammatory and antioxidant effects.

Caffeic acid (CA) is a component that has antioxidant, anti-inflammatory, and anti-amyloidogenic properties that make it a potential treatment against AD. CA exhibits reduced chemical stability as well as limited bioavailability, which hinders its therapeutic potential in vivo. Thus, CA-encapsulated liposomes were designed to enhance its action. These liposomes were conjugated with Tf on their surfaces to facilitate their transport across the BBB. The optimized Tf-modified liposomes were observed to have adequate drug encapsulation performance and stability over a period of two months [59].

Gallic acid (GA) is a compound that, in recent years, has been shown to have strong anti-amyloidogenic properties. Stealth liposomes modified with Tf have been created for the targeted delivery of GA to the brain. These liposomes displayed an average diameter of 130 nm, low polydispersity index values, and a zeta potential that remained neutral. Furthermore, they allowed the continuous release of GA over a period of five days and showed physical stability for a month in storage. Moreover, GA-loaded Tf-functionalized liposomes showed a good interaction with Aβ1-42 monomers, which resulted in a 56% reduction in the number of fibrils produced and a slowing down of Aβ monomer-to-oligomer and oligomer-to-fibril transitions. Additionally, almost 30% of the produced Aβ fibrils were disaggregated by the NPs. According to the results, Tf-functionalized liposomes may be an effective way to deliver GA to the brain for AD treatment [60].

Osthole (Ost), a coumarin molecule with promising therapeutic benefits for AD, has shown neuroprotective properties against Aβ oligomer-induced neurotoxicity in mice; nevertheless, its effectiveness is limited by its low BBB permeability, restricted bioavailability, and solubility. A study used a thin-film hydration technique to produce transferrin-modified osthole liposomes (Tf-Ost-Lip) in order to overcome these difficulties. Tf-Ost-Lip increased intracellular absorption in human brain endothelial cells (hCMEC/D3) and APP-SH-SY5Y cells, suggesting improved drug transport across the BBB. Furthermore, Tf-Ost-Lip had a protective effect on APP-SH-SY5Y cells. In vivo pharmacokinetics and brain tissue distribution analysis also indicated that Tf-Ost-Lip increased Ost accumulation in the brain and prolonged the drug circulation time. Additionally, Tf-Ost-Lip improved Ost’s therapeutic effects on AD-related pathology in APP/PS-1 mice, which included lowering the deposition of Aβ plaques and preventing oxidative stress, neuroinflammation, and apoptosis. According to the study’s findings, Tf-Ost-Lip provides an appealing method for treating AD because of its ability to induce therapeutic effects in vivo and increase drug delivery across the BBB. Despite promising results, further research is required to address key issues such as sterilization methods, stability, encapsulation efficiency, and cytotoxicity [61].

The capacity of Tf to penetrate the BBB can also be improved by specific modifications. Based on previous discoveries on the neuroprotective effects of Pep63, a small peptide, multifunctional liposomes called transferrin-Pep63 liposomes (Tf-Pep63-Lip) have been developed to target both Aβ oligomers and fibrils. These liposomes, loaded with neuroprotective Pep63 and modified with Tf for BBB targeting, were successful in reducing the levels of Aβ in the hippocampus and improving cognitive deficits in AD mice. The combination of phosphatidic acid (PA) and Pep63 within Tf-Pep63-Lip showed synergistic effects. The liposomes not only captured Aβ oligomers or fibrils but also facilitated microglial chemotaxis for clearance. Moreover, Tf-Pep63-Lip inhibited Aβ1-42 aggregation, disaggregated fibrils, and rescued NMDA receptor trafficking, which is fundamental to synaptic plasticity. There were no side effects reported with the therapy, indicating that it was safe to use under the experimental conditions [62].

Tf-conjugated liposomes have emerged as a promising and well-tolerated combination treatment drug for early AD, as they are effective in targeting distinct types of Aβ that are linked to different pathological phases. Nonetheless, further exploration of their therapeutic and adverse effects in clinical trials is essential.

### 5.2. ApoE–Liposomes

Liposomes conjugated with ApoE and phosphatidic acid (PA) have also been studied. ApoE is a glycoprotein that can be found in four different isoforms, including ApoE2, ApoE3, and ApoE4, and contributes to many cellular functions, such as the regulation of synaptic function and the maintenance of BBB integrity [63]. Moreover, ApoE allows the transport of various lipids, such as cholesterol, into the CNS and plasma. ApoE contains a peptide sequence that allows its trafficking across the BBB, while PA is a molecule that exhibits high-affinity binding to Aβ plaques within the brain. Aβ plaques have both positive and negative charges; therefore, PA, which is negatively charged due to the carboxyl group, can interact with the amino terminus of β-amyloid, while ApoE, which has positively charged amino acids, can interact with the negatively charged regions of β-amyloid. As a result, these liposomes can both penetrate the BBB and target Aβ amyloid plaques [64].

A biomimetic ApoE-reconstituted high-density lipoprotein nanocarrier (ANC) constructed from recombinant ApoE and synthetic lipids has been used to target Aβ plagues and improve BBB crossing. An ANC containing the model drug α-Mangostin (α-M), which is known to reduce Aβ fibrils and oligomers, showed therapeutic effectiveness in an AD animal model by decreasing amyloid plaque levels, alleviating microglial activation, and rescuing memory issues. The study also showed a markedly improved absorption of the ANC in brain endothelial cells and an increased brain delivery efficiency in vivo, indicating its potential for treating AD [65].

Both ApoE and PA were shown to modulate calcium concentrations in human cerebral microvascular endothelial cells (hCMEC/D3). Specifically, liposomes modified with an ApoE-derived peptide (mApoE) and PA (mApoE-PA-LIP) were shown to increase the concentration and duration of ATP-induced intracellular Ca^2+^ in cultured astrocytes and hCMEC/D3 cells through the activation of metabotropic purinergic receptors (P2Y). These receptors have been shown to have a protective effect on neuroinflammatory processes. Thus, their activation by liposomes conjugated with ApoE and PA can prevent vasoconstriction caused by Aβ peptides and reduced cerebral blood flow (CBF) [66]. PA- and ApoE-conjugated liposomes were also shown to improve the protective effects of the phytochemicals quercetin and rosmarinic acid against Aβ-mediated cytotoxicity in a rat model of AD [67].

Another study has shown the importance of the synergistic effect of PEGylation and ApoE3 coating on liposome delivery. Rivastigmine hydrogen tartrate (RHT), an important drug for managing AD, shows poor bioavailability due to its hydrophilic nature and significant first-pass metabolism. Khairnar et al. formulated ApoE3-coated RHT-loaded liposomes (ApoE3-RHT-LPS) using DSPE-PEG to improve brain uptake and enhance Aβ clearance. In comparison to pure RHT, the resulting liposomes revealed an increase in brain absorption and a decreased distribution to the liver, indicating an extended drug half-life and circulation time. Furthermore, the formulation was adjusted for increased entrapment efficiency, decreased particle size, and a decreased polydispersity index (PDI). According to stability studies, the formulation does not significantly change in terms of size, PDI, zeta potential, or entrapment efficiency over a period of three months. Overall, the combination of ApoE3-RHT-LPS and PEGylation has a synergistic impact on liposome delivery, and it also lays a solid basis for future research on improving brain uptake and blood circulation time in AD treatment [68].

Phosphatidylcholine (PC) liposomes conjugated with the tripeptide glutathione (GSH) and ApoE (GSH-ApoE-PC) were studied for the delivery of curcumin, quercetin, epigallocatechin gallate, and rosmarinic acid in an in vitro model of the BBB. PC shows specific binding to Aβ, while GSH interacts with the endothelium through carrier-mediated transcytosis, increasing BBB penetration. The addition of stearic acid to the liposome membrane increased the drug release duration, entrapment efficiency, and stabilization. GSH-ApoE-PC liposomes increased BBB permeability, suggesting their potential for enhanced drug delivery to the brain [69].

### 5.3. Magnetite Nanoparticles (MNPs)

Magnetite nanoparticles (MNPs) are nanoparticles with modified surfaces that can contain organic polymers or inorganic elements, either metals or oxides. MNPs consist of a magnetic core, a protective coating, and functional groups that determine their reactivity, solubility, and stability. Active compounds can be attached to the surface or dissolved in their coating. MNPs can be used as vehicles for drug delivery, as they show increased biocompatibility and chemical stability and low toxicity. Furthermore, drug delivery can be regulated in response to an external magnetic field. Once MNPs enter the bloodstream, their superparamagnetic property allows them to move to the target tissues in response to the external magnetic field, where they release the drugs. The nature of the components on the MNP surface determines their magnetic response and therefore their pharmacokinetics. The magnetic forces exerted on the particles compete with the forces of the blood particles, thus determining their target specificity [70,71]. Due to their mode of action and increased target selectivity, they can potentially reduce the required doses and side effects of drugs [70].

A drawback of MNPs, however, is that they tend to aggregate in vivo [72]. To avoid aggregation, MNPs are conjugated to various polymers, such as PEG [73]. MNPs also show limited cell penetration, particularly when transporting materials with low membrane-crossing rates. This problem can be resolved by encapsulating MNPs in liposomes to generate magnetoliposomes (MLPs), which can merge with cell membranes to achieve high delivery rates and cellular absorption without affecting the activity of the functional compounds [9,74]. Another limitation of this approach is that using a constant magnetic force can cause MNPs to aggregate in blood vessels, leading to obstruction. Electromagnetic actuators can be used to generate a high-gradient magnetic field, which prevents MNP aggregation [75]. There is evidence that using electromagnetic guidance improves BBB penetration compared to a constant magnetic force. Electromagnetic-guided MNPs loaded with osmotin, a plant-derived protein with protective effects against neuronal apoptosis, led to a greater reduction in Aβ levels and tau phosphorylation and improved memory function in Aβ_1-42_ -treated rats compared to free osmotin [76].

PEG-conjugated MNPs carrying a specific siRNA on their surface were also shown to inhibit the expression of the β-site APP-cleaving space enzyme 1 (BACE1) gene, a promoter of β-amyloid formation, in HFF-1 fibroblasts, without causing cytotoxicity [77]. These findings highlight the potential of using MNPs as drug delivery vehicles in AD.

## 6. Future Directions and Conclusions

Liposomes, a biocompatible and flexible drug delivery system, have the potential to carry out the targeted delivery of bioactive molecules across the BBB, which prevents access to the brain for around 98% of potential neuropharmaceuticals. Liposomes can incorporate hydrophilic or lipophilic/hydrophobic therapeutic agents, with hydrophilic drugs entrapped in the aqueous core and lipophilic compounds in the hydrophobic region of the lipid bilayer. Various types of therapeutic agents can be attached to the surface of liposomes, creating a targeted multidrug delivery system relevant for treating multifactorial diseases [37].

The clinical translation of liposome-assisted drug delivery platforms has encountered challenges despite significant research efforts. These platforms, although showing therapeutic advantages, face obstacles in pharmaceutical manufacturing, government regulations, and intellectual property issues. Quality assurance concerns, such as scalability and reliability, impact large-scale production, affecting costs and evaluation processes. While conventional liposome production methods have been successful, complexities arise with the addition of surface modifications, leading to increased production costs and challenges in assessing pharmacokinetics and toxicology. Collaboration between academia and industry is crucial for regulatory improvements, establishing a framework conducive to the effective evaluation and translation of nanoformulations into clinical practice [78].

Achieving high drug loading while maintaining stability, ensuring efficient drug delivery to target tissues, and addressing immunogenicity issues are also critical concerns. PEGylation, a common strategy for evading the reticuloendothelial system, may encounter limitations such as the accelerated blood clearance phenomenon. Recent studies report novel coating polymers, such as polydopamine, which has similar bioavailability and efficacy but reduced immunogenicity compared to PEG [79,80]. Scale-up challenges in manufacturing, evaluating long-term safety, and establishing cost-effectiveness also pose hurdles. Clinical trials, regulatory requirements, and patient variability contribute to the complexity. Overcoming these challenges requires concerted efforts from researchers, clinicians, and regulatory bodies to advance liposomal therapies toward safe and effective clinical applications [78].

In the face of a growing global prevalence of brain disorders, particularly AD, there is an urgent need for improved therapeutic strategies. Liposomal formulations, initially explored for cancer treatment, offer a promising avenue for delivering drugs efficiently to the brain. Advances in liposomal formulations are required to improve accuracy and reduce potential side effects. Drawing inspiration from successful cancer therapies like LinTT1 peptide-conjugated liposomes, including the FDA-approved Doxil^®^, we envision the development of tailored formulations for Alzheimer’s treatment. Doxil stands out as a PEG-coated, stealth liposome formulation of DOX, recognized by the FDA as the pioneering nanodrug-based formulation. Another notable example is Depocyt, an FDA-approved liposome formulation of cytarabine designed for treating patients with lymphomatous meningitis, a dangerous complication of lymphoma. Recent studies also highlight the potential of fluorescence-labeled liposomes as diagnostic tools enabling the early diagnosis of AD and, therefore, directing tailored therapies [81].

The continued success of liposomal technologies underscores their potential advantages, including a reduction in drug requirements. As we embark on this transformative era, further research into liposomal applications for Alzheimer’s therapy appears to be a promising option, offering hope for more targeted and effective therapies in the field of neurodegeneration [17].
